# A Life of Service: Lawrence Williams (Larry) Berkley

**DOI:** 10.1120/jacmp.v16i3.5787

**Published:** 2015-05-08

**Authors:** Edward J. Grant

**Affiliations:** ^1^ Medical Physics Department CARTI Inc. PO Box 55050 Little Rock AR 72215

The *JACMP* has lost a dear friend, Larry Berkley. Among his many other services to the medical physics community, Larry was the Chair of the JACMP Journal Business Management Committee for almost ten years, first under the ACMP, and then under the AAPM. He leaves a heritage of knowledge, integrity, and passion for open‐access publishing.

Larry and I go back to the 1969 summer “Rat Camp” at Georgia Tech. I have known him longer than any other medical physicist. I thank Joe Grant for this tribute to Larry's life and legacy. – Michael D. Mills, Editor‐in‐Chief

**Figure 1:**
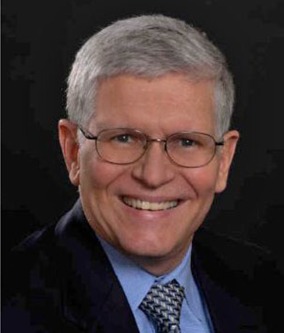


## A LIFE OF SERVICE

Lawrence Williams (Larry) Berkley lived more in his brief 64 years than most of us could manage in two lifetimes. Besides his service to the medical physics community, he was also an accomplished musician, pilot, golfer, sailor, tennis player, EMT volunteer, school board member and president, husband, father and grandfather.

He was born Feb 19, 1951, in Dallas, Texas, to Howard and Elizabeth (Williams) Berkley and grew up in Columbia, Missouri. He graduated from Hickman High School in 1969, where he was known as a top‐notch trumpet player. In his senior year he was featured, along with his private trumpet teacher, then the interim trumpet professor at Mizzou, in a Vivaldi Concerto for 2 Trumpets. His band director, John Patterson, years later said that “Larry played so well he could not tell any difference in Larry's tone and dexterity from that of his professional teacher.”

Larry was drawn to the field of medical physics when he was in his first semester as an undergraduate at Georgia Tech in 1969. His brother, Dick Berkley, was diagnosed with papillary thyroid cancer and treated with a total thyroidectomy and then promptly thereafter with 200 millicurie radioactive iodine. Larry told Dick that this was one of the defining moments for him in choosing medical physics as a career path, along with his natural curiosity and interest in the way the sciences can be used to enhance and even save lives. Today Dick is a 46‐year cancer survivor and an accomplished percussionist with the Austin‐based Texas Symphony.

Larry earned both his B.S and M.S. degrees in physics from the University of Missouri in the early 70s. He moved to the Houston area and began his career with the Radiological Physics Commission (RPC) at M.D. Anderson Cancer Center, where he served for six years; then two more years as the Chief Physicist at Memorial City General Hospital in Houston. Larry began his service to Central Arkansas Radiation Therapy Institute (CARTI) in 1984 as Chief of Medical Physics; at the time the department consisted of two physicists and two dosimetrists at one CARTI location in Little Rock. Before he retired in 2013, as Vice‐President of Physics, Engineering and Information Services, he had guided CARTI through a steady expansion that included the addition of five more sites in central and north Arkansas, with a physics staff of five staff physicists, two medical physics residents, and ten dosimetrists.

A strong believer in education, he helped to develop the CARTI RTT school's physics curriculum; helped develop a clinical dosimetry certificate program (of which I was an early participant and beneficiary); served as director of the Medical Dosimetry Certification Board for six years; served as Director of the ABMP for one year; and finally served as the first director of the CARTI medical physics residency program, which was one of the first purely clinical and professional residency programs in the USA to gain CAMPEP accreditation in 2011.

His commitment to universal public education and inclusiveness expanded beyond CARTI. He served for ten years on the Little Rock School Board, two of those years as President, and he was a member of the City of Little Rock Racial and Cultural Diversity Commission.

The Little Rock School Board was historically known for being a rather contentious body, which was where Larry's talent as a mediator was a true asset. One of the other members said years later that Larry's service was clearly motivated by his desire to reconcile widely divergent views, and that he was strongly driven to provide the best educational environment for all of the children of the Little Rock District.

I asked Mary Hulett, Larry's stepsister, to provide a comment about her memory of Larry.

“He was the glue that held the family together. When our two families merged in the early 70s, Larry and I immediately became very close. I think mostly because we were the same age. When there was friction between siblings, Larry was there to smooth things over. He was able to mediate and knew the best way to resolve conflicts. He didn't shy from challenges and was always the most intelligent one of all of us. He was wise beyond his years. I would often ask Larry about his opinion on a wide variety of topics just to see what he thought, and I enjoyed our discussions. We kept in touch through the years, even though our paths diverged regionally. This was mostly because of Larry's efforts. He knew the importance of family. I was often amazed at how calmly he would handle the raising of his children, in spite of teen angst or growing pains. He was truly a great mediator. I think this was one of his strengths as a Board of Education member.”

When asked to provide a comment to a local newspaper that would summarize Larry's outlook, his brother Dick stated: “He was good at bringing people together, and seeing past people's differences in order to get them to a common ground to solve problems.”

The two years after his “retirement” from CARTI, until his passing on March 7, 2015, were a time of unstoppable activity for him. “He loved to be doing something all the time,” said his daughter, Megan Daniels. “He wasn't one of those people that retired and didn't do anything. He had a willingness to work for others without reward. He worked to make society better, doing what was right and fair. He taught his children the importance of that.”

In his new community of Fairfield Bay, Arkansas, he developed a whole new life and friendships and, of course, community activism — becoming an avid golfer, tennis player, and EMT volunteer squad driver. He continued to work as a medical physics consultant with Mid‐America Physics. And his old love of music came back, so he started a community brass ensemble. With all of his activities, I began to wonder if he ever slept. A fond memory — I also had a background in music, so we shared that interest, and once even teamed up on trumpet duets in his church. It was with some amusement, and great affection, that I remember about a year ago, getting texts from him around midnight, asking if I could recommend any good low brass players for his group!

I had the honor to work for Larry for 18 years as a member of the CARTI physics staff. I remember him as a very smart and principled physicist, and one of the most fair‐minded leaders I have ever known. He was also a good friend and a fine mentor; I will always be grateful that he gave me my opportunity in this wonderful career. He was universally respected at CARTI, in the Little Rock community, and in the national physics community. His life was truly a life of service and he is missed by all of us.

Larry is survived by his wife, Barbara, and their children, Megan (Joe and Ellie Kate) Daniels, Kimberly (Scott and Alex) Kaczenski, Timothy Berkley and Justin Wesslels; siblings Dutch and Dick Berkley, Mary, Brad and Bill Hulett; and his favorite mother‐in‐law, Grace Minton.

## Supporting information

Supplementary Material

Supplementary Material

