# Supplementary figures and images for: A Life of Service: Lawrence Williams (Larry) Berkley
Source: J Appl Clin Med Phys. 2015 May 8;16(3):9–11. doi: 10.1120/jacmp.v16i3.5787 (PMC5690137; doi:10.1120/jacmp.v16i3.5787)

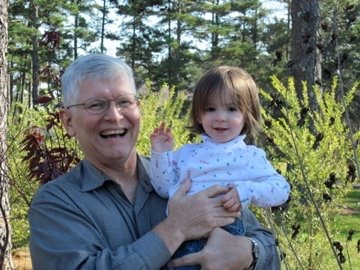

Supplement: Supplementary file 1 — Supplementary Material [file ACM2-16-009-s001.docx]

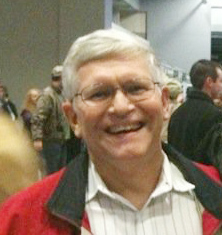

Supplement: Supplementary file 2 — Supplementary Material [file ACM2-16-009-s002.jpg]
